# Antidiabetic treatment restores adiponectin serum levels and APPL1 expression, but does not improve adiponectin-induced vasodilation and endothelial dysfunction in Zucker diabetic fatty rats

**DOI:** 10.1186/1475-2840-12-46

**Published:** 2013-03-18

**Authors:** Peter M Schmid, Markus Resch, Christian Schach, Christoph Birner, Guenter A Riegger, Andreas Luchner, Dierk H Endemann

**Affiliations:** 1 Klinik und Poliklinik für Innere Medizin 2, Franz-Josef-Strauss Allee 11, University of Regensburg, Regensburg, 93042, Germany

**Keywords:** Adiponectin, APPL1, AdipoR, eNOS, Insulin, Metformin, Diabetes mellitus type 2

## Abstract

**Background:**

Adiponectin is able to induce NO-dependent vasodilation in Zucker lean (ZL) rats, but this effect is clearly alleviated in their diabetic littermates, the Zucker diabetic fatty (ZDF) rats. ZDF rats also exhibit hypoadiponectinemia and a suppressed expression of APPL1, an adaptor protein of the adiponectin receptors, in mesenteric resistance arteries. Whether an antidiabetic treatment can restore the vasodilatory effect of adiponectin and improve endothelial function in diabetes mellitus type 2 is not known.

**Methods:**

During our animal experiment from week 11 to 22 in each case seven ZDF rats received an antidiabetic treatment with either insulin (ZDF+I) or metformin (ZDF+M). Six normoglycemic ZL and six untreated ZDF rats served as controls. Blood glucose was measured at least weekly and serum adiponectin levels were quantified via ELISA in week 11 and 22. The direct vasodilatory response of their isolated mesenteric resistance arteries to adiponectin as well as the endothelium-dependent and -independent function was evaluated in a small vessel myograph. Additionally, the expression of different components of the adiponectin signaling pathway in the resistance arteries was quantified by real-time RT-PCR.

**Results:**

In ZDF rats a sufficient blood glucose control could only be reached by treatment with insulin, but both treatments restored the serum levels of adiponectin and the expression of APPL1 in small resistance arteries. Nevertheless, both therapies were not able to improve the vasodilatory response to adiponectin as well as endothelial function in ZDF rats. Concurrently, a downregulation of the adiponectin receptors 1 and 2 as well as endothelial NO-synthase expression was detected in insulin-treated ZDF rats. Metformin-treated ZDF rats showed a reduced expression of adiponectin receptor 2.

**Conclusions:**

An antidiabetic treatment with either insulin or metformin in ZDF rats inhibits the development of hypoadiponectinemia and downregulation of APPL1 in mesenteric resistance arteries, but is not able to improve adiponectin induced vasodilation and endothelial dysfunction. This is possibly due to alterations in the expression of adiponectin receptors and eNOS.

## Background

Adiponectin is a 30-kDa adipokine, which is predominantly and abundantly secreted form adipose tissue [1-3]. Unlike serum levels of other adipokines, levels of adiponectin characteristically correlate negatively with obesity and its comorbidities [2,4-6]. Therefore hypoadiponectinemia serves as a marker of adipose tissue dysfunction and the metabolic syndrome [7,8]. Adiponectin exerts insulin-sensitizing and antiinflammatory actions in liver and muscle cells [9,10] and has vasoprotective effects [11-13]. Adiponectin binds at the cellular level to two specific transmembrane receptors, adiponectin receptor 1 (AdipoR1) and adiponectin receptor 2 (AdipoR2) [14,15], which provokes an intracellular activation of the 5`-AMP-activated protein kinase (AMPK) and PPAR-alpha [16]. APPL1 (adaptor protein, phosphotyrosine interaction, PH domain and leucine zipper containing 1) is the first described adaptor protein for the adiponectin receptors [17] and seems to play an important role in the intracellular signal transmission from the receptors to AMPK and PPAR-alpha as well as in the crosstalk with the insulin signaling pathway [18-20]. In contrast, its protein isoform, APPL2 (adaptor protein, phosphotyrosine interaction, PH domain and leucine zipper containing 2), seems to act as a negative regulator of adiponectin signaling [21].

In the last years several studies could prove that adiponectin causes NO production in endothelial cell cultures by binding to its receptors and a consecutive activation of APPL1, AMPK and endothelial nitric oxidase (eNOS) [22-25]. Recently, we [26] could show that adiponectin indeed provokes a NO-dependent vasodilation of mesenteric resistance arteries in nondiabetic Zucker lean (ZL) rats. Additionally, this vasodilation was diminished in their diabetic littermates, the Zucker diabetic fatty (ZDF) rat, which also exhibited endothelial dysfunction. As possible pathological mechanism we found hypoadiponectinemia in ZDF rats and a downregulation of APPL1 in their mesenteric arteries at mRNA level. For this reason, we concluded that a decreased adiponectin signaling through hypoadiponectinemia and a reduced sensitivity to adiponectin is at least in part responsible for endothelial dysfunction in diabetes mellitus type 2. This hypothesis was supported by other studies demonstrating endothelial dysfunction in hypoadiponectinemia and an improvement of endothelial function by replenishment of adiponectin levels [27-29].

In this study we wanted to test, whether an antidiabetic treatment with either insulin or metformin can improve endothelial dysfunction and restore vasorelaxation to adiponectin in ZDF rats. Further, we wanted to detect effects on the expression levels of adiponectin receptors, APPL1, APPL2 and endothelial nitric oxidase (eNOS) via real-time RT-PCR.

## Methods

### Animal experiments

The study was approved by the local committee on animal research and is in accordance with the “Guide for the Care and Use of Laboratory Animals” published by the US National Institutes of Health. Six male Zucker lean (ZL) rats (fa/-) and twenty male Zucker diabetic fatty (ZDF) rats (fa/fa) were studied from an age of 11 weeks until an age of 22 weeks. Of the ZDF rats, six were kept as untreated controls throughout the whole experiment, seven were treated with insulin subcutaneously (ZDF+I) and the remaining seven animals were treated with metformin solved in tap water (ZDF+M). In the insulin group blood glucose was measured every second day after 6 hours fasting (ACCU-CHEK Sensor, Roche GmbH, Mannheim, Germany). Treatment target was to reach nearly normal blood glucose levels. Therefore the insulin dose was adjusted to the last blood glucose value prior to the planned insulin administration. Blood glucose in the metformin group was quantified twice a week and metformin dose was increased from initially 300 up to 600 mg/kg/day as the maximum tolerable concentration without severe toxic side effects for rats [30]. Blood glucose in untreated ZDF and ZL rats was measured weekly. Animals were individually housed on a 12-hour dark/12-hour light cycle. They were fed a Purina 5008 rat chow containing 23% protein, 6.5% fat, 58.5% carbohydrates, 4% fiber and 8% ash. Rats received tap water ad libitum. At baseline (week 11) and in the last week (week 22) serum levels of adiponectin were measured (B-Bridge International, Inc., Mountain View, USA) via ELISA according to the instructions of the manufacturer. Systolic blood pressure as well as heart rate was assessed every two weeks by tail cuff method using an automated cuff inflator-pulse detection system (CODA2 Multi-Channel, Computerized, EMKA TECHNOLOGIES, Paris, France). Body weight was measured weekly. At the end of the experiment animals were sacrificed and the mesenteric vasculature was dissected for preparation of small resistance arteries.

### Preparation and study of small resistance arteries

As described previously [26], a third-order branch from the mesenteric artery of the different investigated rats was prepared and mounted on a pressure myopraph (DMT, Aarhus, Danmark). First, the vessels media thickness and the lumen diameters were measured. Then, after precontraction with norepinephrine (10^-5^ mol/L), endothelium-dependent and independent relaxation was assessed with acetylcholine (ACh) (10^-9^-10^-4^ mol/L) and sodium nitroprusside (SNP) (10^-5^-10^-2^ mol/L), respectively. Also, the endothelial vasodilator function of adiponectin was quantified after precontraction with norepinephrine (10^-5^ mol/L) by a stepwise administration of globular adiponectin in cumulative concentrations from 0.03125 to 0.5 μg/ml) (AXXORA GmbH, Lörrach, Germany). The NO-dependency of the observed vasodilatory response to adiponectin was tested by an additional preincubation and application of L-nitroarginine-methyl-ester (L-NAME) (10^-5^ mol/L), an inhibitor of the endothelial NO synthase (eNOS).

In all experiments the vasodilation of the vessels was calculated as percentage after a maximal precontraction with 10^-5^ mol/L norepinephrine using the following formula: vasodilation (%) = (D_X_-D_NE_)/(D_R_-D_NE_)x100. Thereby D_X_ is the actual measured diameter at a given concentration of adiponectin, ACh or SNP. D_NE_ is the diameter under precontration with norepinephrine, and D_R_ is the resting diameter of the vessel.

### Expression of adiponectin receptors, APPL1, APPL2 and eNOS

The expression of the adiponectin receptors, AdipoR1 and AdipoR2, the adaptor proteins, APPL1 and APPL2, as well as the expression of eNOS in the small arteries was measured by using a real-time RT-PCR. Therefore, same calibre branches of the mesenteric artery were collected and cleaned from adipose and connecting tissue. Total RNA was extracted using RNeasy kit (Quiagen, Hilden, Germany) according to the instructions of the manufacturer. For first-strand cDNA synthesis, 1 μg total RNA was reverse transcribed with 1U MMLV Reverse Transcriptase, 1 μg Random Primer, 1 mM deoxynucleotide triphosphate mixture, 1 μl recombinant RNasin® ribonuclease inhibitor and transcription buffer with 5 mM MgCl_2_ in a final volume of 10 μl (all from Promega, Mannheim, Germany). The reaction mixture was incubated at 37°C for 60 min, followed by heat inactivation of the enzyme at 95°C for 5 min. After cooling on ice for 5 min, the cDNA was stored at − 20°C. In parallel, 1 μg total RNA was processed without reverse transcription to control for contamination with genomic DNA. Real-time RT-PCR detection of AdipoR1, AdipoR2, APPL1, APPL2 and eNOS was carried out using the AbiPrism 7900 TaqMan (Applied Biosystems, Foster City; CA, USA). Beta-actin was used as housekeeping gene for normalization. The TaqMan probes for AdipoR1, AdipoR2, eNOS, iNOS, and for beta-actin were purchased from Applied Biosystems (Foster City, USA). Primers for APPL1 and APPL2 bridging at least one intron were designed and purchased from MWG-Operon (Ebersberg, Germany) as described previously [26].

### Statistical analysis

From the assessed biometric data, the levels of serum parameters and the relative mRNA expressions the means of every measurement for each animal group were calculated. The groups were compared using one-way ANOVA with Holm-Sidak or Fisher LSD method as post hoc test. Outliers were detected and excluded with the Nalimov test. The curves created from the measurements in the small vessel myograph were compared using two-way ANOVA for repeated measurements with Holm-Sidak method as post hoc test. All data are presented as means ± SEM. A p-value of <0.05 was considered to be significant.

## Results

### Biometric data and blood glucose levels of ZL and ZDF rats

Table 1 shows the biometric data of different animal groups exemplarily at the beginning and the end of the experiment. In week 12 ZL rats were significantly lighter than diabetic ZDF rats. Under the fed high-caloric diet in the course of the animal experiment ZL rats gained about 25% of weight and it was equal to untreated and metformin-treated ZDF rats in the following weeks, which also showed a slight weight gain during the experiment. In contrast, insulin-treated ZDF rats rapidly and excessively increased their body weight by about 60% until week 22 due to anabolic effects of insulin. Consecutively, their weight was significantly higher than in the other animal groups at the end of the experiment. Considering the systolic blood pressure, significant differences could be detected only in week 12 with a higher blood pressure in ZL in comparison to ZDF rats. In the following weeks the blood pressure equalized between the groups. During the whole experiment heart rate was significantly higher in ZL rats than in untreated and metformin-treated ZDF rats. Insulin-treated ZDF rats showed higher heart rates in comparison to the other diabetic animal groups only in week 12.

**Table 1:** Biometric data and blood glucose levels

**Parameters**	**ZL**	**ZDF**	**ZDF+I**	**ZDF+M**	**p**
	**(n = 6)**	**(n = 6)**	**(n = 7)**	**(n = 7)**	
Body Weight at Week 12 [g]	333 ± 10*	367 ± 11	376 ± 6	377 ± 10	0.001
Body Weight at Week 22 [g]	415 ± 14	410 ± 15	603 ± 10†	425 ± 14	0.001
SBP at Week 12 [mmHg]	151 ± 6§	118 ± 3	128 ± 5	125 ± 6	0.002
SBP at Week 22 [mmHg]	137 ± 6	129 ± 7	125 ± 3	136 ± 2	0.425
Heart Rate at Week 12 [bpm]	595 ± 16#	471 ± 26	543 ± 12¥	475 ± 18	0.001
Heart Rate at Week 22 [bpm]	549 ± 14⍑	460 ± 17	489 ± 12	465 ± 23	0.023
Blood Glucose at Week 11 [mg/dl]	77 ± 4~	245 ± 15	239 ± 8	226 ± 9	<0.001
Blood Glucose at Week 22 [mg/dl]	75 ± 3Φ	259 ± 17	106 ± 10¶	231 ± 24	<0.001

Abbreviations: *ZL* Zucker lean rats, *ZDF* Zucker diabetic fatty rats, *ZDF+I* Zucker diabetic fatty rats treated with insulin, *ZDF+M* Zucker diabetic fatty rats treated with metformin, *SBP* systolic blood pressure. p p-value for one-way ANOVA.

Post hoc testing: * p < 0.001 vs. ZDF, ZDF+I and ZDF+M. † p < 0.001 vs. ZL, ZDF+I and ZDF+M. § p < 0.001 vs. ZDF, p = 0.002 vs. ZDF+M, p = 0.005 vs. ZDF+I. # p < 0.001 vs. ZDF and ZDF+M. ¥ p = 0.011 vs. ZDF, p = 0.009 vs. ZDF+M. ⍑ p = 0.005 vs. ZDF, p = 0.007 vs. ZDF+M. ~ p < 0.001 vs. ZDF, ZDF+I and ZDF+M. Φ p < 0.001 vs. ZDF and ZDF+M. ¶ p < 0.001 vs. ZDF and ZDF+M.

**Figure 1:**
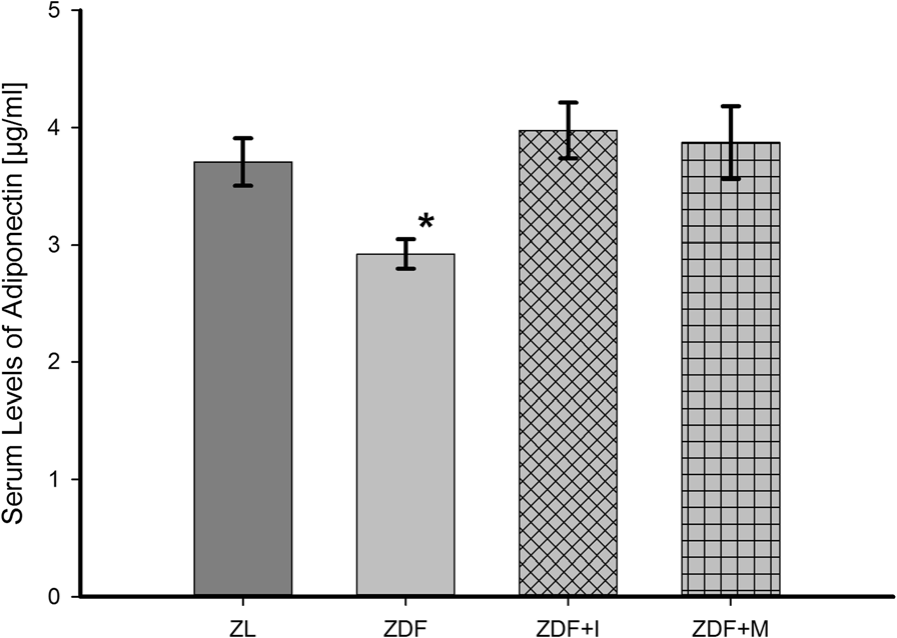
**Serum adiponectin levels in week 22.** Untreated ZDF animals show significantly reduced levels of adiponectin, which are restored by insulin or metformin treatment. One-way ANOVA p = 0.042. Post hoc testing: * p = 0.040 vs. ZL, p = 0.008 vs. ZDF+I, p = 0.018 vs. ZDF+M.

Table 1 also depicts blood glucose levels in week 11 and 22. As expected, ZDF rats exhibited already at an age of 12 weeks significantly elevated blood glucose levels in terms of diabetes mellitus type 2. In contrast, ZL rats had normal blood glucose during the experiment. Concurrently, it got obvious that treatment with metformin even in highest tolerable doses had no relevant effect on blood glucose levels. Only treatment with insulin could decrease blood glucose to normal levels even though they were still slightly higher than in ZL rats. Adiponectin serum levels were similar at the beginning of the experiment (ZL 4.1 ± 0.1 μg/ml; ZDF 3.8 ± 0.4 μg/ml; ZDF+I 4.0 ± 0.5 μg/ml; ZDF+M 4.2 ± 0.3 μg/ml. p = 0.929). In the next weeks adiponectin levels decreased only in untreated ZDF rats, while they were stable in nondiabetic ZL rats and insulin- as well as metformin-treated ZDF rats. This resulted in a significant hypoadiponectinemia of ZDF rats in comparison to all other animal groups in week 22 (Figure 1).

### Relaxation response to adiponectin

**Figure 2:**
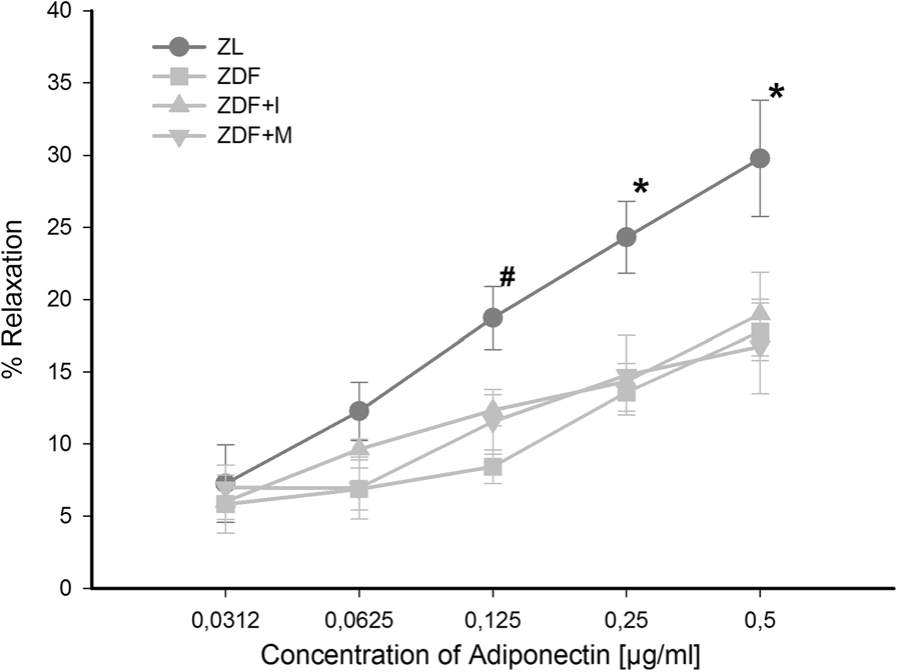
**Dose-dependent relaxation of resistance arteries to cumulative doses of adiponectin after precontraction with norepinephrine.** Adiponectin-induced vasodilation is restricted in untreated ZDF animals and remains unchanged by insulin or metformin treatment. Two-way ANOVA for repeated measurements: * p = 0.002 vs. ZDF and ZDF+I, p = 0.001 vs ZDF+M; # p = 0.002 vs ZDF.

**Figure 3:**
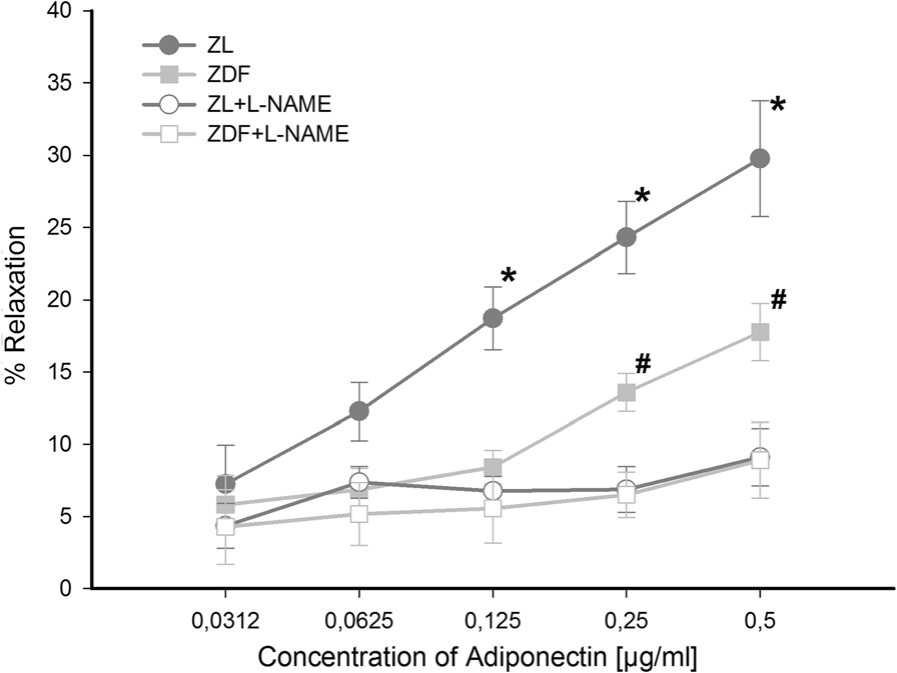
**Dose-dependent relaxation of resistance arteries to cumulative doses of adiponectin with and without L-NAME after precontraction with norepinephrine exemplary for ZL and ZDF rats.** Adiponectin-induced vasodilation is nearly totally blunted by L-NAME indicating NO-dependency of vasodilation. Two-way ANOVA for repeated measurements: * p < 0.001 vs. ZL+L-NAME; # p < 0.001 vs. ZDF+L-NAME.

The vasodilation response of small resistance arteries to adiponectin was tested after precontraction with norepinephrine (Figure 2). We found a dose dependent vasodilatory response for adiponectin especially in normoglycemic ZL rats. Maximum vasodilation was reached at a concentration of 0.5 μg/ml adiponectin with 30 ± 4% in ZL rats. In contrast, vasodilation in ZDF rats was clearly blunted, but a slight vasodilation could also be detected with a maximum vasodilation of 18 ± 2% at highest used concentrations of adiponectin. Of notice, treatment with insulin or metformin did not improve vasodilatory response to adiponectin in small resistance arteries (maximum vasodilation: ZDF+I 19 ± 3%; ZDF+M 17 ± 3%). Only differences between ZL and ZDF rats irrespective of treatment were statistically significant. To test, whether the observed vasodilatory response of the small arteries to adiponectin is NO dependent, small arteries were treated with L-NAME, an inhibitor of eNOS, additionally to adiponectin. Thereby, the initially noted vasodilatory effect of adiponectin was completely blunted (Figure 3). This indicates that the vasodilatory effect of adiponectin is mediated by NO.

### Relaxation response to acetylcholine and sodium nitroprusside

**Figure 4:**
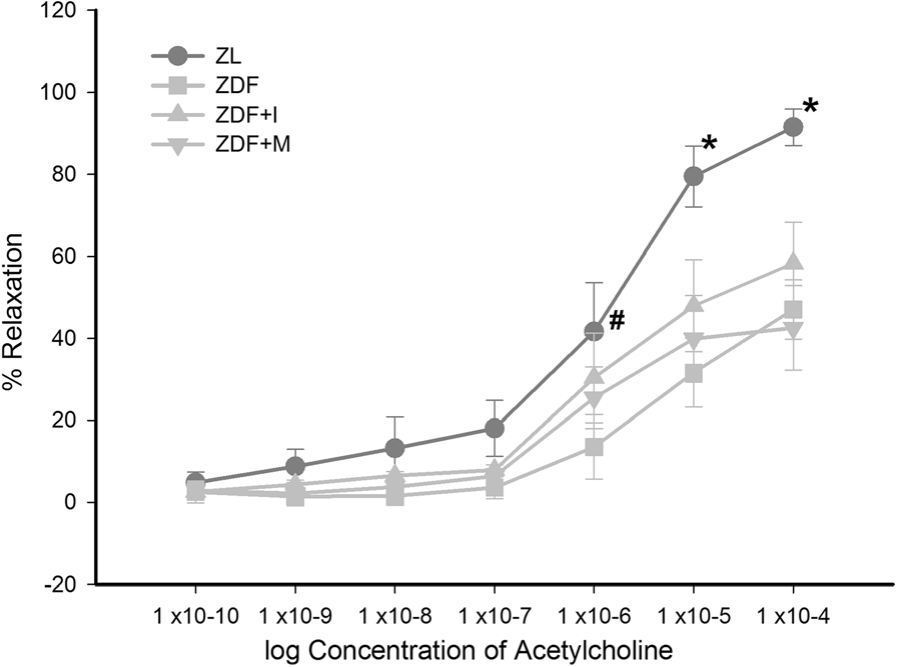
**Dose-dependent relaxation of resistance arteries to cumulative doses of acetylcholine (ACh) after precontraction with norepinephrine.** Acetylcholine-induced vasodilation is diminished in ZDF animals indicating endothelial dysfunction, which can´ t be improved by insulin or metformin. Two-way ANOVA for repeated measurements: * p < 0.001 vs. ZDF, ZDF+I and ZDF+M; # p = 0.004 vs. ZDF.

Endothelial function was assessed by stimulation of small resistance arteries with ACh after precontraction with norepinephrine (Figure 4). We found a clear dose-dependent vasodilation with a maximum relaxation of 92 ± 4% for ZDL rats at a concentration of 10^-4^ mol/L ACh. This was diminished in all diabetic animals regardless of treatment, indicating endothelial dysfunction in these animals (maximum vasodilation ZDF 47 ± 7%; ZDF+I 58 ± 10%; ZDF+M 43 ± 10%).

Endothelium-independent vasodilation was assessed by treatment with SNP (data not shown). Again, vasodilation was dose-dependent and tended to be lower in diabetic rats, but the difference between ZL and untreated ZDF rats was not statistically significant.

### Expression of adiponectin receptors, APPL1, APPL2, and eNOS in mesenteric resistance arteries

The expression of different essential components of the adiponectin signaling pathway was measured in the mesenterial arteries by real-time RT-PCR to reveal possible causes of a different vasorelaxation response to adiponectin. Thus we performed an expression analysis for the adiponectin receptors (AdipoR1 and AdipoR2), the adiponectin receptor adaptor proteins (APPL1 and APPL2), and eNOS.

**Figure 5:**
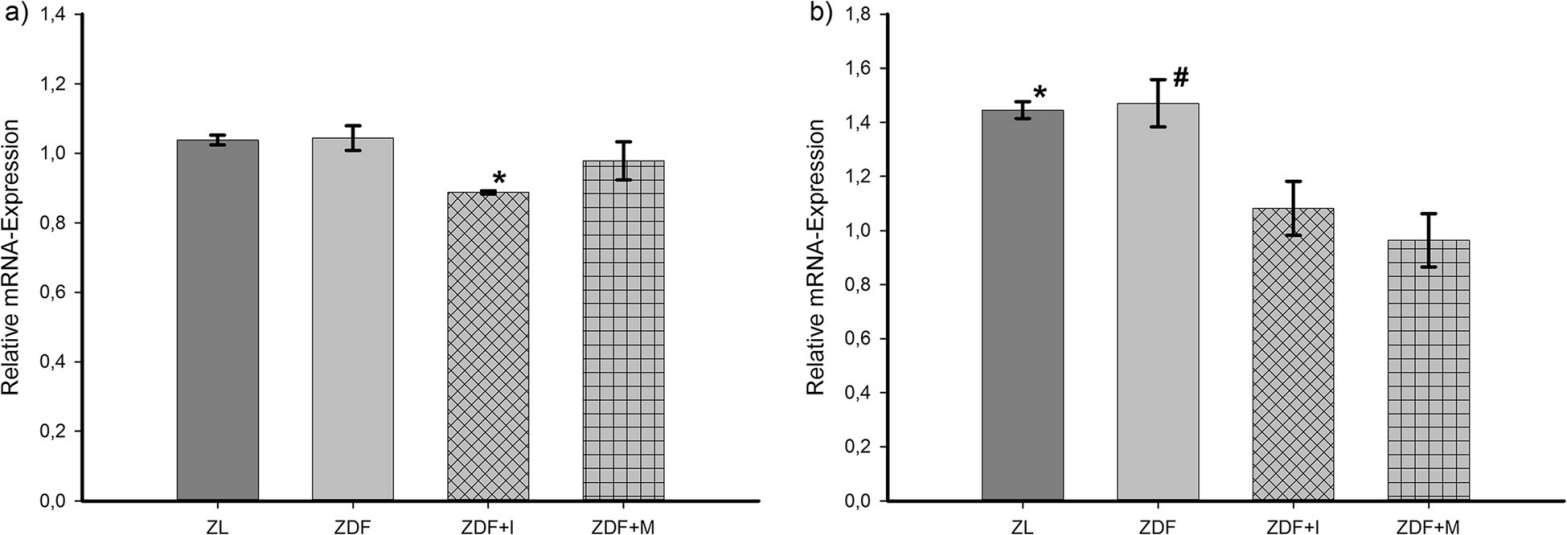
**Relative mRNA-expression of adiponectin receptors in small resistance arteries. a**) Expression of AdipoR1. Insulin causes a significant downregulation of AdipoR1 expression. One-Way ANOVA p = 0.024. Post hoc testing: * p = 0.007 vs. ZL, p = 0.008 vs. ZDF+M **b**) Expression of AdipoR2. Insulin or metformin treatment causes a significant downregulation of AdipoR2 expression. One-way ANOVA p < 0.001. Post hoc testing: * p = 0.006 vs. ZDF+I, p < 0.001 vs. ZDF+M; # p = 0.005 vs. ZDF+I, p < 0.001 vs. ZDF+M.

**Figure 6:**
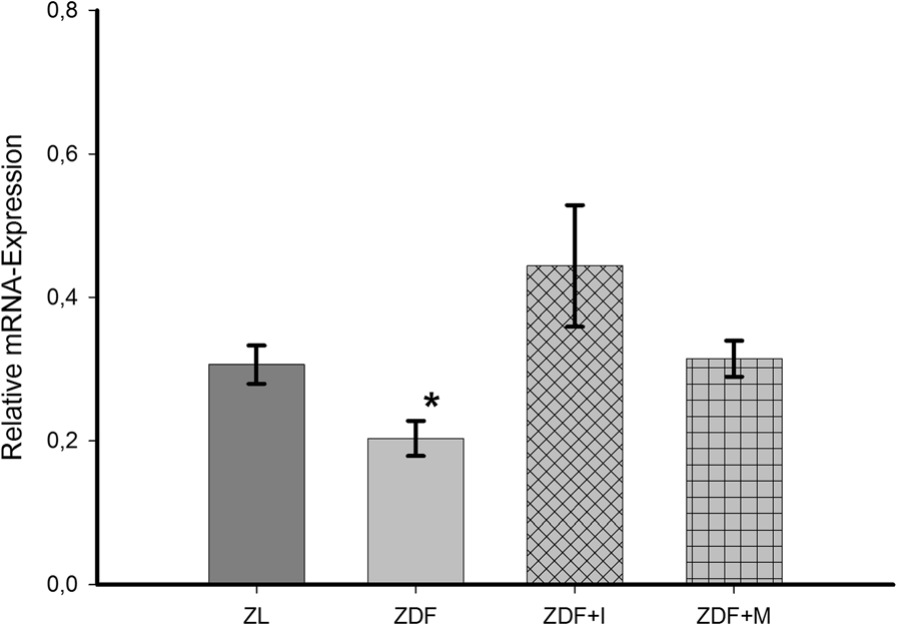
**Relative mRNA-expression of APPL1 in small resistance arteries.** APPL1 expression is significantly reduced in untreated ZDF animals and expression levels are restored by insulin or metformin treatment. One-way ANOVA p = 0.03. Post hoc testing: * p = 0.028 vs. ZL, p = 0.044 vs. ZDF+I, p = 0.017 vs. ZDF+M.

We found that AdipoR1 (Figure 5a) is expressed in a similar amount in ZL and ZDF rats. Insulin provoked a significant decrease in AdipoR1 expression in comparison to untreated ZDF rats, while in metformin-treated animals AdipoR1 expression remained unchanged. Likewise, the expression of AdipoR2 (Figure 5b) was similar in ZL and ZDF rats, but treatment with either insulin or metformin caused a significant decrease. Overall APPL1 expression (Figure 6) was lowest in untreated ZDF rats. Its expression was significantly increased by metformin and insulin. Also, in ZL rats APPL1 expression was significantly higher than in untreated ZDF rats. However, no significant differences could be detected for the expression of APPL2. Its expression was highest in insulin-treated (ZDF+I 1.1 ± 0.12) and lowest in untreated and metformin-treated ZDF rats (ZDF 0.89 ± 0.05; ZDF+M 0.79 ± 0.05), but the differences in both cases did barely not reach the significance level (p = 0.09 vs. ZDF; p = 0.057 vs. ZDF+M).

**Figure 7:**
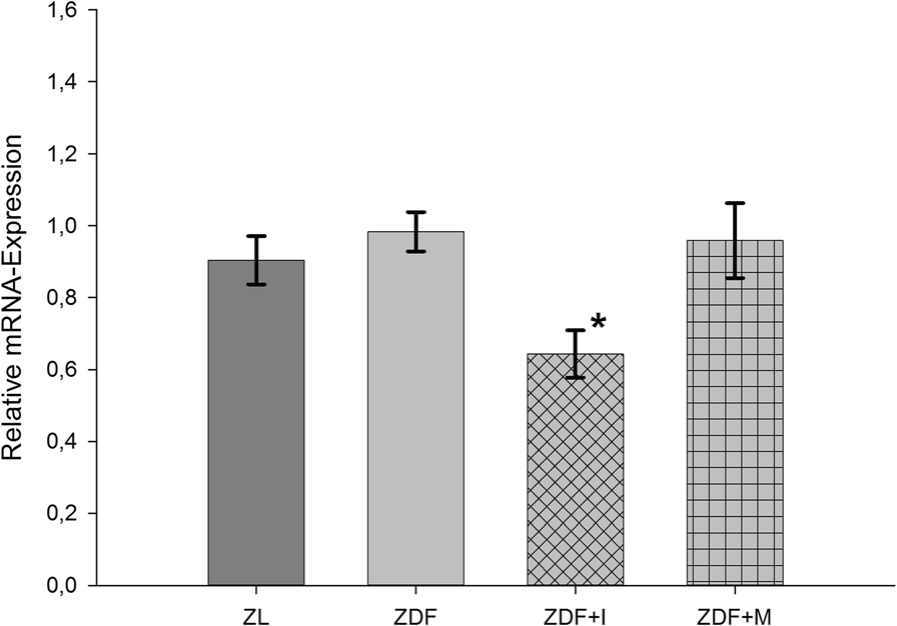
**Relative mRNA-expression of eNOS in small resistance arteries.** Insulin causes a significant downregulation of eNOS expression. One-way ANOVA p = 0.047. Post hoc testing: * p = 0.048 vs. ZL, p = 0.01 vs. ZDF, p = 0.016 vs. ZDF+M.

eNOS expression in small resistance arteries (Figure 7) was similar high in ZL and untreated ZDF rats. Treatment with insulin significantly decreased the expression of eNOS, while treatment with metformin did not affect the expression of eNOS.

## Discussion

The major findings of our present study are: (1) an antidiabetic treatment with insulin or metformin inhibits the development of hypoadiponectinemia and downregulation of APPL1 in ZDF rats, but (2) both treatments are not able to improve adiponectin-induced vasodilation and endothelial dysfunction; (3) treatment with insulin is able to control blood glucose levels in ZDF rats, but (4) causes a decrease of AdipoR1, AdipoR2 and eNOS expression in small mesenteric resistance arteries; (5) treatment with metformin even in highest tolerable doses does not decrease blood glucose levels in ZDF rats and (6) reduces expression of AdipoR2 in mesenteric arteries.

### Effect of antidiabetic treatment on adiponectin-induced vasodilation and endothelial function

In our recent study [26] we found that adiponectin provokes a NO-dependent vasodilation of mesenterial resistance arteries in ZL rats, which is diminished in arteries of ZDF rats. The expression analysis of different components of the adiponectin signaling pathway showed a significant downregulation of APPL1 in mesenteric arteries of ZDF rats. In addition ZDF rats exhibited hypoadiponectinemia and endothelial dysfunction. Therefore, we concluded that hypoadiponectinemia itself and a reduced intracellular adiponectin signaling are partly responsible for endothelial dysfunction in diabetes mellitus type 2. This hypothesis was supported by a study of Ouchi et al. [29], who found that adiponectin-knockout mice exhibit endothelial dysfunction and another study of Cao et al. [27], in which a replenishment of adiponectin in knock-out mice normalized endothelial function. Similar Deng et al. [28] could show that endothelial dysfunction in high-fat diet fed rats was ameliorated by an in vitro incubation of aortic rings with globular adiponectin in high doses. Therefore interventions directed to increase adiponectin levels in diabetes mellitus type 2 are possibly able to improve endothelial function. Against our expectations in the present study an antidiabetic treatment with insulin or metformin could not improve the vasodilatory effect of adiponectin and endothelial function in diabetic ZDF rats (Figure 2+4), although both restored adiponectin levels and also increased the expression of APPL1 in ZDF rats to levels of nondiabetic ZL rats (Figure 1+6). Thereby, it has to be mentioned that these results of course only apply to our ex vivo setting and that we can`t make a statement to possible in vivo effects of the tested antidiabetic treatment.

In insulin-treated animals the reason is probably the observed downregulation of both adiponectin receptors (Figure 5) and eNOS (Figure 7), which with their localization at the beginning and end of the adiponectin signaling pathway are the main regulators of adiponectin sensitivity and NO-production. Therefore, the reduced expression of the adiponectin receptors and eNOS might diminish the benefical effects of increased adiponectin serum and APPL1 expression levels. Additionally, the increase of APPL1 expression might be counteracted by the complementary increase of APPL2 expression, even if not statistically significant. The fact that an early treatment with insulin in diabetes mellitus type 2 does not improve endothelial function is in line with a recent study in human, in which flow-mediated dilation remained unchanged after initiation of insulin therapy [31].

For metformin-treated animals our study suggests two possible reasons for the absent improvement of vasodilation. On the one hand the reduced expression of AdipoR2 (Figure 5b) and on the other hand the uncontrolled blood glucose levels. Thereby, decreased expression of AdipoR2 may at least in part reduce adiponectin sensitivity resulting in decreased eNOS activation and NO production. The fact that metformin is not able to control blood glucose in ZDF rats was already observed in another study, since ZDF rats develop insulin deficiency between an age of 14 and 21 weeks [32]. An uncontrolled diabetes mellitus may cause endothelial dysfunction through additional mechanisms apart from adiponectin signaling. Accordingly, a study in another rat model of diabetes mellitus type 2 showed an improvement of endothelial function in aortic rings after metformin therapy, but this effect was associated with a significant improvement of blood glucose levels [33]. However, in another experiment metformin therapy improved endothelial function independent of a blood glucose lowering effect, but this study was performed in streptozotocin-induced diabetic rats, a model of diabetes mellitus type 1 [34].

### Effect of antidiabetic treatment on adiponectin levels and expression of components of the adiponectin signaling pathway

As mentioned above, both antidiabetic treatments inhibited the development of hypoadiponectinemia during the animal experiment (Figure 1).

Generally, the effect of insulin on adiponectin serum levels is contentious. In vitro, insulin inhibits the expression of adiponectin at mRNA level [35], but then it leads to an increased adiponectin secretion from adipocytes [36,37]. In vivo, there is a good documented association of hypoadiponectinemia with insulin resistance and diabetes mellitus type 2 [38], but the effect of insulin therapy on serum levels of adiponectin is not well studied so far. A study in five healthy male volunteers showed a decrease of adiponectin levels during a hyperinsulinemic euglycemic clamp [39]. Another study with insulin treatment in a murine model of obesity and diabetes mellitus type 2, the db/db mice, suggested no impact on adiponectin levels [40]. A recent study indicates an elevation of adiponectin levels in a hyperinsulinemia rat model [41]. In this respect, our study supports new evidence for a positive effect of insulin therapy on adiponectin levels despite of an increase in body weight.

For the effect of metformin on adiponectin levels there are more studies available. In vitro metformin increased the expression and secretion of adiponectin in human adipose tissue samples [42]. However, results of studies in humans with metformin treatment for different diseases showed mostly no effect on adiponectin serum levels [43-48], but it also exists evidence for an adiponectin-raising effect [49-51]. In this context our animal study supports a benefical effect of metformin on adiponectin levels.

Considering the adiponectin receptors, we found under insulin treatment a significantly decreased expression of AdipoR1 and AdipoR2 compared to untreated animals (Figure 5). On this so far data from an in vitro study exist showing a reduced expression of both adiponectin receptors in skeletal and vascular smooth muscle cells by insulin [52]. Another recent study also revealed that an insulin infusion in a rat model decreased expression of AdipoR1 in skeletal and myocardial muscle [41]. This effect seems to be mediated by a direct repressive effect of insulin on the promoter activity of AdipoR1 [53]. Our data match these studies and further support and expand these findings to the vasculature. The effect of metformin on adiponectin receptor expression was so far studied in adipose tissue, skeletal muscle and liver of ZDF rats [32]. In this experiment a significant increase for both receptors in skeletal muscle was detected and our study now expands these findings with expression data for small mesenteric resistance arteries showing a decreased expression of AdipoR2.

To our knowledge, with the present study we provide first data on the effect of an antidiabetic treatment on the expression of APPL1 and APPL2 showing that insulin as well as metformin is able to increase the expression of APPL1 in resistance arteries of ZDF rats, while APPL2 expression remained unchanged (Figure 6).

In respect to eNOS, we found that insulin treatment significantly reduced its expression in mesenteric arteries (Figure 7). This goes with findings of Zanetti et al., who showed that an overexpression of eNOS in diabetic animal model can be attenuated by insulin treatment [54]. However, another study in ZDF rats found no effect of an insulin therapy on eNOS expression in the aorta [55]. Metformin treatment did not alter eNOS expression in our experiment, which is in line with studies in other animal models of diabetes mellitus [56].

However, our expression analyses are performed with real-time RT-PCR and therefore only reflect expression at mRNA level, since second order mesenteric arteries of rats do not yield enough protein for a reliable western blot analysis.

## Conclusion

ZDF rats as a model of diabetes mellitus type 2 are characterized by hypoadiponectinemia, a reduced expression of APPL1 and a decreased response of their mesenteric arteries to adiponectin and acetylcholine in terms of endothelial dysfunction. In our present study, we explored the effect of an antidiabetic treatment with insulin or metformin on their endothelial function and the expression of different components of the adiponectin signaling pathway. We found that both treatments restored adiponectin serum levels and expression of APPL1 in small mesenteric resistance arteries, but were not able to improve endothelial function. This is possibly due to a reduced expression of AdipoR1, AdipoR2 and eNOS under insulin treatment and a reduced expression of AdipoR2 and uncontrolled blood glucose under metformin therapy in our animal model. Since the phenotype of type 2 diabetes mellitus in the ZDF animal model is based on a homozygous mutation in the leptin receptor, our results can only be applied with restrictions to humans. In addition, it can't be excluded that our results are at least in part influenced by the genetic difference between ZL and ZDF rats.

## Competing interests

The authors declare that they have no competing interests.

## Authors’ contributions

PS, MR and DE designed research; PS, MR, CS, CB and DE performed research; GR and AL revised the manuscript critically for intellectual content; PS and DE analyzed data; PS and DE wrote the paper. All authors have read and approved submission of the final manuscript.
